# Recent Advances in Tactile Sensing Technology

**DOI:** 10.3390/mi9070321

**Published:** 2018-06-25

**Authors:** Minhoon Park, Bo-Gyu Bok, Jong-Hyun Ahn, Min-Seok Kim

**Affiliations:** 1 Center for Mechanical Metrology, Korea Research Institute of Standards and Science, 267 Gajeong-ro, Yuseong-gu, Daejeon 34113, Korea; minhoon825@kriss.re.kr (M.P.); wcsp@kriss.re.kr (B.-G.B.); 2 School of Electrical and Electronic Engineering, Yonsei University, 50 Yonsei-ro, Seodaemun-gu, Seoul 03722, Korea; ahnj@yonsei.ac.kr

**Keywords:** soft robotics, tactile sensing, robot-assisted surgery, artificial skin, human tactile perception

## Abstract

Research on tactile sensing technology has been actively conducted in recent years to pave the way for the next generation of highly intelligent devices. Sophisticated tactile sensing technology has a broad range of potential applications in various fields including: (1) robotic systems with tactile sensors that are capable of situation recognition for high-risk tasks in hazardous environments; (2) tactile quality evaluation of consumer products in the cosmetic, automobile, and fabric industries that are used in everyday life; (3) robot-assisted surgery (RAS) to facilitate tactile interaction with the surgeon; and (4) artificial skin that features a sense of touch to help people with disabilities who suffer from loss of tactile sense. This review provides an overview of recent advances in tactile sensing technology, which is divided into three aspects: basic physiology associated with human tactile sensing, the requirements for the realization of viable tactile sensors, and new materials for tactile devices. In addition, the potential, hurdles, and major challenges of tactile sensing technology applications including artificial skin, medical devices, and analysis tools for human tactile perception are presented in detail. Finally, the review highlights possible routes, rapid trends, and new opportunities related to tactile devices in the foreseeable future.

## 1. Introduction

Human perception relies on information gathered from the surrounding environment using the five senses of sight, hearing, taste, smell, and touch. There is little doubt that humans could have not survived without these senses. Considerable attempts have been made to incorporate tactile sensing capabilities into highly intelligent robotic systems by equipping such systems with sophisticated and delicate sensors that emulate human skin, as sensory information with a number of modalities could improve the accuracy of robot interaction with unstructured environments [1,2,3,4,5,6,7,8]. Furthermore, researchers are starting to employ tactile sensing technology in prosthetic hands and arms to provide tactile feedback to patients with amputated limbs [9,10]. Such developments could lead to improved user-friendly experiences by mitigating phantom limb pain, increasing perception levels, which helps patients feel as though a prosthetic is a part of the body, and by relieving the stress resulting from the control of robotic prosthetics based on visual and auditory feedback only.

Recent advances in tactile sensing technology have benefited greatly from the development of new materials and special structural engineering techniques in combination with existing physical principles. Newly developed scalable, ultrathin conducting or semiconducting materials such as nanomembrane single crystal silicon [11,12,13], graphene [14,15,16,17], and molybdenum-disulfide (MoS_2_) [18] are considered as the materials of choice for tactile sensors due to the mechanical properties required for electronic skin: flexibility, stretchability, and conformability. On the other hand, new types of flexible tactile sensors that employ unique sensing structures such as crack shapes [19,20,21,22] and interlocked structures [23,24,25,26,27] with piezoresistive or capacitive transduction schemes have been developed to achieve higher pressure sensitivity that exceeds that of humans. 

This review begins with an introduction to skin physiology in order to understand the underpinning mechanisms of human tactile sensing, which is important in the development of artificial biomimetic tactile sensors. This section is followed by a review of recent achievements in the development of tactile sensors, which is divided into sections according to critical aspects including sensitivity, shear (or slip) sensing, methods in matrix-type sensor arrays, and materials. In the following sections, several examples of tactile sensing applications are introduced, including tactile perception analysis, multi-functional e-skin (electronic skin) for humanoid robots, and robotic surgery. The conclusion summarizes the obstacles that should be addressed, as well as perspectives. Readers are also referred to [28,29,30,31] for a comprehensive review of state-of-the-art tactile sensing technologies. 

## 2. Fundamental Theories of Human Skin

**Figure 1:**
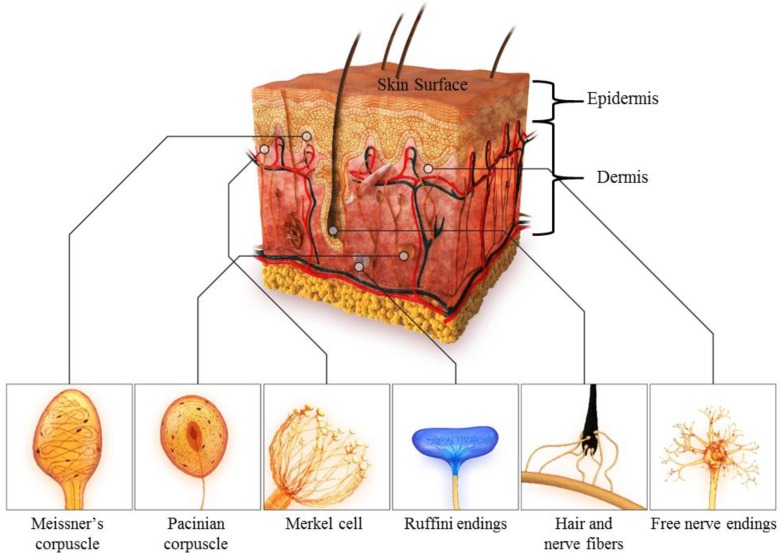
Description of mechanoreceptors in human hand skin.

In the glabrous or hairless skin of human fingertips, four main types of tactile receptors or cutaneous mechanoreceptors are embedded in the dermis at different depths: Merkel cells, Meissner corpuscles, Ruffini endings, and Pacinian corpuscles [28,32,33,34,35,36]. Each receptor responds exclusively to specific mechanical stimuli, and the response depends on the mechanical properties of the skin. The characteristics of the above mechanoreceptors related to tactile perception are summarized in Table 1. The four main receptors can be categorized into two groups according to adaptation rate: rapid adapting (RA) units and slow adapting (SA) units. SA-type receptors produce a time-invariant output for sustained static stimuli, whereas RA-type receptors respond to dynamic stimuli. In addition, each group includes type I and type II receptors, according to the size of the receptive field and density, as shown in Figure 1. Type I receptors are located close to the surface of the skin, and have small receptive fields, whereas Type II receptors are located deeper in the dermis and have larger receptive fields. The SA-I mechanoreceptor, the Merkel cell, is responsible for static pressure distribution. The SA-II mechanoreceptor, the Ruffini corpuscle, detects skin stretch and slips at the fingertips. The Meissner corpuscle RA-I-type mechanoreceptor exhibits the highest sensitivity for low-frequency (10–50 Hz) vibrations and is responsible for light touch (tapping), grip control, and texture discrimination. The Pacinian corpuscles or Lamellar corpuscles, which are RA-II-type receptors, are optimized to detect vibrations elicited by relative motions between the skin and an object in a frequency range between 200–300 Hz, and play a critical role in the perception of surface texture. These four mechanoreceptors that provide various modalities of tactile sense enable humans to perform precise and delicate tasks with their hands than any robotic system can do.

The mechanoreceptors in our skin convey tactile information to our brain through nerve fibers by generating pulse trains with frequencies that are proportional to the magnitude of the stimuli. Such a digital transduction scheme allows the transmission of signals with greater accuracy and reliability to the brain. If tactile sensing devices are integrated into a prosthetic, the devices should be equipped with appropriate electronic circuits for tactile feedback, which transform analog into digital signals that are acceptable to the human sensory system. Although certain studies have demonstrated flexible tactile sensors that mimic the transduction mechanism of SA-type mechanoreceptors by integrating an oscillator with a pressure sensor [37], there are still challenges to overcome to develop electronic skin that is on par with human skin. Examples of such limitations include the integration of a large number of sensing elements in limited space (e.g., at fingertips) and sensor-addressing problems (i.e., how to read data from each discrete sensor).

## 3. Requirements for Tactile Sensing

Developing so-called “skin-like” tactile sensors could be a challenging task. The artificial skin should have high sensitivity, fast response, and durable and high spatial resolution with multimodal (e.g., pressure, slip, temperature, vibration) sensing capability. Among them, we selected three critical requirements that have been advanced by several research groups, which include sensitivity, sensor-addressing, and shear (or slip) sensing. High sensitivity is required to measure small forces (or pressure), as humans’ skin can detect light touch, of which pressure is approximately down to 5 kPa [38]. Sensor addressing is another important issue, as mentioned in the above section, to read data from array sensors arranged in the form of an *N* (number of rows) × *M* (number of columns) array. This layout of a matrix form can minimize the number of wires for addressing *N* + *M*, but it has greater cross-talk problems, because it allows parasitic conduction paths. Therefore, the output of an individual array element is contaminated by other elements on the same array. To remove or reduce such parasitic conduction, a layout scheme that connects a diode (passive type) or a transistor (active type) to each sensing element in the series is required. The third issue is slip sensing. Sometimes, it is desirable for robotic grippers to measure tangential shear forces. For example, when a robotic gripper grasps an object, it must apply a perpendicular force high that is enough to produce a tangential force that exceeds the weight of the object. Therefore, slip sensing is essential, unless the gripper can tolerate considerable overpressure. The following subsections will outline recent achievements in the tactile sensing technologies with a focus on these issues. 

### 3.1. High Sensitivity

**Figure 2:**
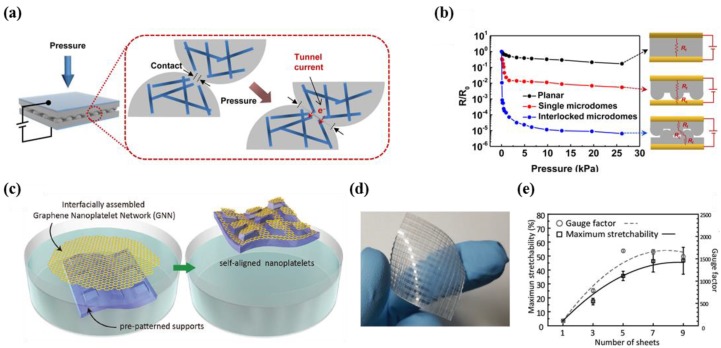
(**a**) Schematic illustration demonstrating the working mechanism of the sensor. Tunnel current occurs when the pressure is applied to the device; (**b**) output curves showing the difference in pressure sensitivity according to the sensor structure (reprinted with permission for Figure 2a,b from Ref. [25] Copyright 2014 American Chemical Society); (**c**) schematic illustration demonstrating the sensor fabrication method by using the liquid-driven transfer technique; (**d**) image of the skin-conformal sensor array; (**e**) gauge factor and maximum stretchability according to the number of transferred layer (reprinted with permission for Figure 2c–e from Ref. [39] Copyright 2017 John Wiley and Sons).

In order to enhance sensitivity, many researchers have conducted intensive studies on the development of sensors using micro or nanoscale surface structures of various metal materials [23,24,25,26,27]. Park et al. studied interlocked microdome arrays that employ carbon nanotube (CNT) composite-based rubber. This sensor exhibited a uniquely high sensitivity performance (pressure: 15.1 kPa^−1^, minimum detection value: 0.2 Pa) due to the giant tunneling effect (Figure 2a) [25]. Park et al. explained that the applied strain creates concentrations at the contact region, which induce a tunneling resistance via electron charge. In particular, the interlocked microdome array exhibited outstanding performance compared with planar and single microdome arrays with switching behavior (10^5^) (Figure 2b). Although CNT composite-based rubber in the interlocked microdome array showed non-linear behavior in the output curve, along with typical drift and hysteresis reported in piezocomposite materials, the simple fabrication process and high sensitivity of piezocomposite materials allow for applications such as the detection of the motion of a snail or human breathing.

Park et al. first demonstrated a strain sensor for skin-attachable devices that uses conductive patterns and self-assembled graphene nanoplatelet networks (GNN) [39]. The researchers transferred graphene platelets onto pre-patterned supports in a water-based suspension with an organic solvent to form conductive GNN patterns (Figure 2c). In particular, the organic solvent has a significant role in the process of obtaining self-assembled shapes with large area layers. The behavior was analyzed as a result of Rayleigh–Bernard convection and Marangoni forces. Figure 2d shows a photograph of a highly sensitive capacitive conformal sensor array with a double-sided layer that was produced using this method. In particular, this sensor has a gauge factor of 1697 and high stretchability when five layers of GNN are adopted, despite the decrease in optical transparency (Figure 2e). The ultra-sensitivity is advantageous for applications in the detection of biosignals and spatial distribution, as demonstrated in the research.

**Figure 3:**
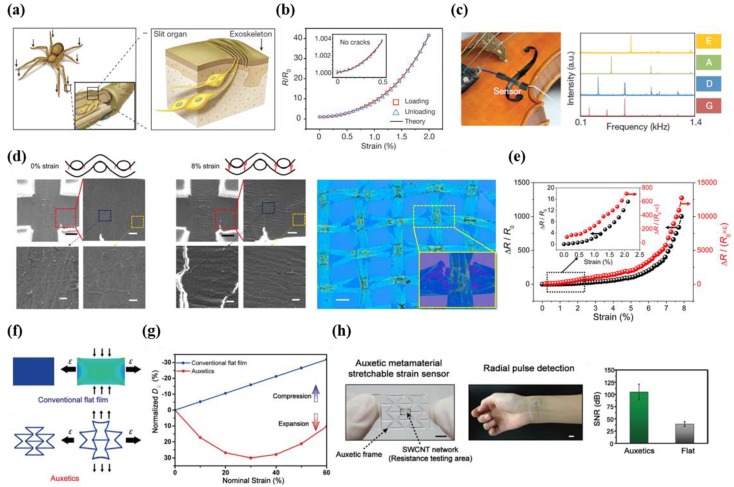
(**a**) Image of the special spider organ for detecting external stimuli (left) and the magnified image of the organ (right); (**b**) the relative output resistance change versus strain, including theoretical data; the inset shows the results of no cracks; (**c**) image of crack sensor onto violin for detection of sound wave (left). The intensity versus frequency, when Elgar’s ‘Salut d’Amour’ was played (right) (reprinted with permission for Figure 3a–c, from Ref. [20] Copyright 2014 Springer Nature); (**d**) SEM image shows the stretched graphene woven fabric (GWFs) depending on the strain (left). Optical image of the GWF on Si/SiO_2_ substrate (right); (**e**) data showing the relative output resistance under different strain levels. (Reprinted with permission for Figure 3d,e from Ref. [22] Copyright 2015 American Chemical Society); (**f**) finite element modeling (FEM) simulation data including conventional flat film structure and auxetic metamaterial structure under 15% tensile strain; (**g**) the normalized displacement at the transverse direction under different levels of longitudinal tensile strain; (**h**) auxetic strain sensor and human wrist with sensor (left). The signal-to-noise ratio data in case of auxetics and flat sensors (right) (Reprinted with permission for Figure 3f–h from Ref. [40] Copyright 2018 John Wiley and Sons).

A spider is highly sensitive to vibratory signals from its surroundings due to a special organ with a crack-shaped geometry (Figure 3a) [20]. Strain sensors that mimic this unique nanoscale crack structure possess ultra-sensitive output characteristics and mechanical flexibility (Figure 3b). Kang et al. demonstrated a highly sensitive sensor that uses a crack-based principle. The results shown in Figure 3b indicate that the crack-based strain gauge sensor possesses 450-fold higher resistance variation compared with samples without cracks at a strain of 0.5%. The gauge factor was measured to be as high as 2000 at 0–2% strain. This group demonstrated that the nanoscale crack sensor could be placed on the surface of a violin to sense sound wave-induced vibrations (Figure 3c).

Yang et al. described a graphene-based strain gauge sensor based on a crack mechanism [22]. The researchers introduced significant structural innovations such as crisscross-shaped graphene microribbons (GMRs) (Figure 3d). Atmospheric pressure chemical vapor deposition (APCVD) graphene was grown on a crisscross copper mesh to develop a graphene woven fabric (GWF) sensor. The sensor exhibited gauge factors of 500 below 2% strain and 10^4^ above 8% strain (Figure 3e). In addition, the developed GWF strain sensor enables applications in human motion detection, acoustic signal acquisition, and spatially distributed pressure sensing in real time.

Conventional thin films exhibit low sensitivity due to reversal movements in the longitudinal and transverse directions under strain (i.e., Poisson’s effect). Jiang et al. addressed this problem by incorporating auxetic metamaterials in the sensing structure (Figure 3f). Such structure is also capable of providing better stretchability than that of conventional thin films [40]. The results shown in Figure 3g reveal that the estimated sensitivity in gauge factor is approximately 835 at 15% tensile strain, which is a value that represents a 24-fold enhancement in comparison to the sensitivity of conventional thin film-based sensors (~35). The auxetic metamaterial stretchable strain sensor is capable of detecting radial artery pulses in humans while maintaining a high signal-to-noise ratio (SNR) of 104.8 dB, whereas conventional flat film sensors are limited to 39.4 dB (Figure 3h).

### 3.2. Active Matrix Circuitry

A large number of cells are required in a strain sensor array for measurements over a wide area. For example, 62,500 cells exist over a surface area of 1 × 1 cm^2^ in human glabrous skin, which has a spatial resolution of 40 μm. Hence, researchers frequently experience problems such as complex wiring when monitoring pressure distribution from external stimulations. In order to solve such issues, passive matrix strategy, which is an efficient and simple solution, was suggested [41,42,43,44]. However, several challenges still remain in the use of tactile sensors, including large crosstalk between adjacent cells. Hence, the optimal design that has been introduced in the recent literature is active matrix circuitry, which is similarly utilized in display applications [45,46,47,48]. Several researchers have recently conducted studies regarding pressure sensor arrays that utilize the aforementioned effective solution (i.e., active matrix circuitry) and incorporate various structures and materials [48,49,50,51,52,53,54,55,56,57,58,59]. Active matrix circuitry contributed to the development of feasible strain sensors with skin-like functions in the following research achievements. This review highlights the advantages of active matrix circuitry rather than the characteristics of the unusual materials exploited in the studies.

**Figure 4:**
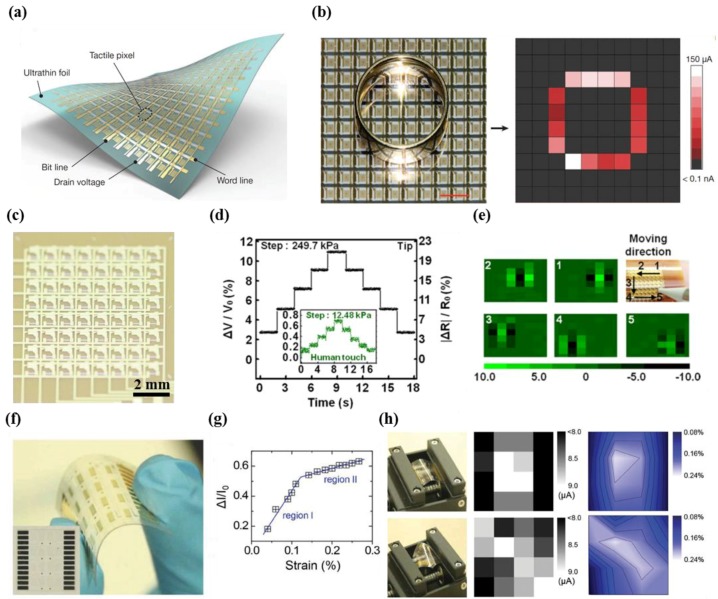
(**a**) Illustration of the tactile sensor array with active matrix circuitry; (**b**) photographic image showing the tactile sensor array (left). The mapping data by pressure of the ring (right) (reprinted with permission for Figure 4a,b from Ref. [49] Copyright 2013 Springer Nature); (**c**) photograph of silicon membrane tactile sensor with active matrix circuitry; (**d**) real-time monitoring of the fractional change in voltage versus the pressure levels in real time; (**e**) data and image showing the movement of stylus tip (reprinted with permission for Figure 4c–e from Ref. [51] Copyright 2015 AIP Publishing); (**f**) photograph showing active matrix array sensor based on the graphene; (**g**) sensitivity performance of graphene transistor (GT) strain sensor including region I and II; (**h**) the image and output data under different bending states (left) (reprinted with permission for Figure 4f–h from Ref. [54] Copyright 2015 John Wiley and Sons).

Kaltenbrunner et al. demonstrated an ultrathin active matrix array with 12 × 12 pixels based on the tactile sensors of conductive pressure sensitive rubber and air-stable high-mobility p-type semiconductor DNTT (dinaphtho[2,3-b:2′,3′-f] thieno[3,2-b]thiophene) (Figure 4a) [49]. Notably, the developed ultrathin organic field-effect transistors (OFETs) had an average mobility of 0.88 ± 13 cm^2^ V^−1^ s^−1^ among all 144 transistors, an on/off ratio of (2.57 ± 0.7) × 10^7^, and low gate leakage currents of less than 100 pA. Due to having superior properties, the developed sensor array is advantageous for mapping the pressure distribution for circular shapes with high sensitivity and precise acquisition (Figure 4b).

Park et al. described a prototype silicon nanomembrane-based tactile sensor of an 8 × 8 array with active matrix circuitry (Figure 4c) [51]. The thin film single-crystal Si transistor possessed superb characteristics, including an on/off ratio of 10^6^, a field effect mobility of 661 cm^2^ V^−1^ s^−1^, and a threshold voltage of 0.5 V. Figure 4d shows that a wide range of pressure measurements can be measured in real time with a minimum detectable pressure of 12.4 kPa, which corresponds to the threshold values of human skin (10–40 kPa). Furthermore, a stable current enables a high switching frequency of as high as 100 kHz to map pressure distribution, as shown in Figure 4e. The transistor array with a high on/off ratio provides low crosstalk (i.e., interference among elements), which leads to improvement in the resolution of tactile images.

Sun et al. reported active matrix sensor arrays that included nanogenerators made of Poly(vinylidenefluoride-*co*-trifluoroethylene) (P(VDF-TrFE)) and coplanar-gate graphene thin film transistors (TFTs) [54]. A device of a 4 × 4 coplanar-gate graphene transistor (GT) array with gold electrodes for reading signals was fabricated on a 2.5 × 2.5 cm^2^ polyethylene terephthalate (PET) substrate (Figure 4f). The calculated gauge factor was 389 in region I (69 in region II), and the minimum detectable strain values were below 0.008% (Figure 4g). The results in Figure 4h indicate that the developed active matrix sensor can be used to visualize two-dimensional strains through quantitative measurements, due to the excellent characteristics of the sensor.

**Figure 5:**
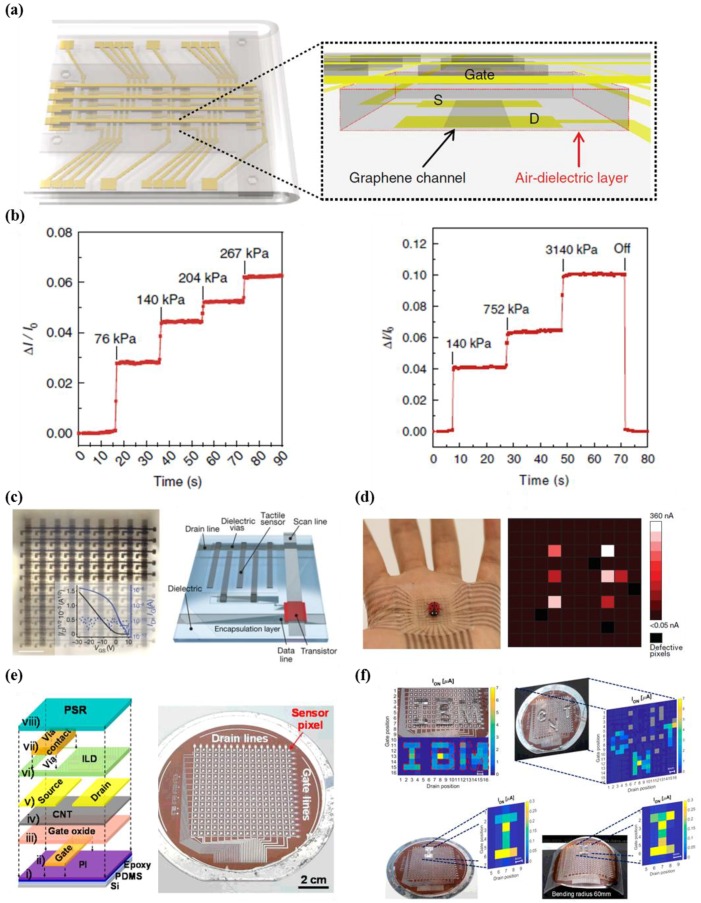
(**a**) Schematic illustration of pressure-sensitive graphene FETs (left). Cross-sectional image unit cell with a graphene channel and an air dielectric layer (right); (**b**) the characteristic of the air dielectric graphene transistors as a function of time at various pressure levels (left: maximum level of 267 kPa, right: maximum level of 3140 kPa) (reprinted with permission for Figure 5a,b from Ref. [52] under CC-BY 4.0 license); (**c**) image of intrinsically stretchable transistor and illustration of unit cell with tactile sensor, electrodes, dielectric, and passivation layer; (**d**) photograph showing the conformal transistor array with ladybug on palm (left), current mapping data resulting from the pressure of the six legs of a ladybug (right) (reprinted with permission for Figure 5c,d from Ref. [58] Copyright 2018 Springer Nature); (**e**) schematic illustration showing fabrication steps of a flexible pressure sensor (left), the photographic image of the active matrix tactile sensor with 16 × 16 array; (**f**) the current mapping data indicating pressure distribution at flat and bending states (radius of 60 mm) (reprinted with permission for Figure 5e,f from Ref. [50] Copyright 2018 American Chemical Society).

A tactile pressure-sensitive sensor based on a graphene field effect transistor (FET) array that uses air dielectric layers was developed by Shin et al. [52]. In particular, a simple folding method was utilized on origami substrates to build local air gaps, which resulted in devices with outstanding characteristics and stability (Figure 5a). The introduced FET showed impressive electrical properties, including mobility values of 212 cm^2^ V^−1^ s^−1^ (p-type) and 96 cm^2^ V^−1^ s^−1^ (n-type) in addition to a wide pressure detection range from 250 Pa to 3 Mpa, as shown in Figure 5b. However, further studies are required, as the large off-current of the developed graphene-based FETs results in limitations of high power consumption and severe crosstalk, according to the literature.

In recent years, Wang et al. used intrinsically stretchable TFT active matrix array sensors as backplanes, while other researchers adopted geometric strategies with rigid active materials [58]. This sensor has 6300 on-board transistors within an area of 4.4 cm^2^ for addressing good conformability and semi-transparency (Figure 5c). Each transistor showed a stable mobility of 0.98 cm^2^ V^−1^ s^−1^. In addition, a 10 × 10 array of resistive tactile sensors (resolution of 2 mm) consisting of interdigitated carbon nanotube (CNT) electrodes of high stretchability facilitate conformal contact onto a human palm (Figure 5d). The researchers successfully achieved the spatial mapping of external strains induced by six conductive legs of an artificial ladybug.

Common drawbacks, such as a relatively low mobility and large operation voltage, should be carefully considered. In this regard, transistors based on carbon nanotube (CNT) materials are advantageous for the active matrix circuitry due to their outstanding electrical properties, high stability in ambient conditions, and insensitivity to the mechanical strain [55,59]. Recently, Nela et al. demonstrated a fully integrated flexible pressure sensor that uses CNT transistor active matrix circuitry with a 16 × 16 array over a large area (4-inch) and pressure-sensitive rubber for e-skin (Figure 5e) [50]. The field-effect carrier mobility was measured as 17.6 cm^2^ V^−1^ s^−1^, in contrast to the small mobility values of organic transistors. By combining CNT transistors and pressure-sensitive rubber, the researchers demonstrated the capability of e-skin to detect objects with arbitrary shape, as shown in Figure 5f. The results show that the sensor is capable of stable operation while maintaining small crosstalk within a small voltage range of 3 V, and is usable on flat and curved surfaces with a radius of curvature of 60 mm.

### 3.3. Shear Force Sensing

Upon interaction with objects or textures, human skin receives multiple tactile inputs including normal forces, shear forces, and vibrations, resulting in the recognition of surface shape, texture, and slip [28,60]. Shear force and vibration-sensing capabilities are essential for determining the coefficient of friction and the fineness of object surfaces, respectively. A common approach used to achieve multiple functionality, including normal and shear forces sensing, involves the adoption of a bump or force-localizing protrusion (FLP) at the center of the unit cell, which enhances sensitivity and enables the detection of information related to surface textures [61,62,63,64,65,66,67,68,69,70,71,72,73].

**Figure 6:**
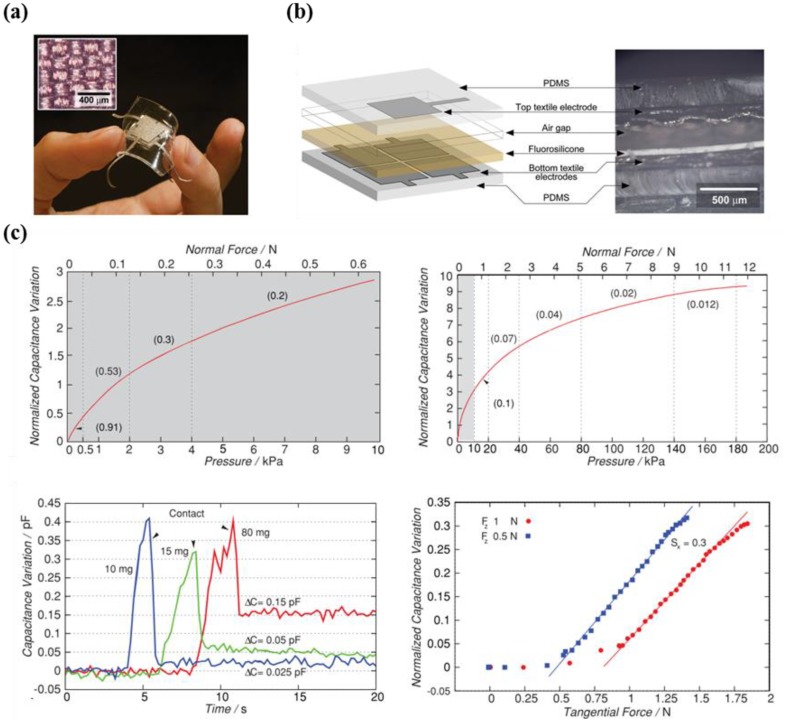
(**a**) Photograph and optical microscopic image indicating the flexible three-axial shear force sensor; (**b**) illustration and SEM image depicting the cross-sectional view of the sensor; (**c**) output characteristics (sensitivity under normal and shear force/the different weight of 80 mg, 15 mg, and 10 mg) of flexible shear force sensor (reprinted with permission for Figure 6a–c from Ref. [68] Copyright 2014 John Wiley and Sons).

Viry et al. fabricated a highly flexible capacitive three-axial force sensor, which consists of conductive fabric electrodes with an intrinsic resistance of 50 mΩ/square, via rapid and economic fabrication strategies (Figure 6a) [68]. The developed sensor includes perpendicular groups of conductive yarns (warp and weft) and an elastomeric material (Figure 6b). Due to the air gaps in fluorosilicone, which was used to form a dielectric layer, the developed sensor possessed outstanding capabilities such as a minimum detection resolution of less than 10 mg and displacement of 8 μm. The introduced three-axial force sensor was measured to have an estimated detection range from 190 kPa to 400 kPa (Figure 6c,d). The researchers expect the developed high performance tactile sensor to be useful in various fields, including highly advanced robotics, wearable devices, biomedical systems, and touch panel systems for the gaming industry.

**Figure 7:**
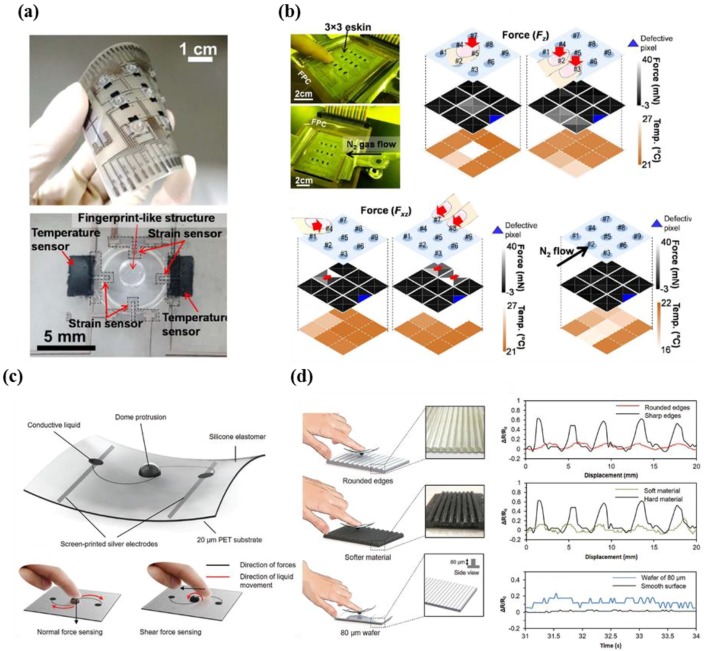
(**a**) Photograph showing the fully fabricated sensor with 3 × 3 array (top) and unit cell (bottom); (**b**) real applications of the device using normal force, shear force, and N_2_ flow (reprinted with permission for Figure 7a,b from Ref. [67] Copyright 2014 American Chemical Society); (**c**) schematic illustration demonstrating the unit cell of the flexible microfluidic tactile sensor, and working principle; (**d**) illustration of texture sensing experiment and related output performances (rounded edges, softer material, and 80 μm) (reprinted with permission for Figure 7c,d from Ref. [72] Copyright 2017 John Wiley and Sons).

Harada et al. developed a strain-engineered three-axis tactile sensor (36 strain gauges) and a temperature sensor (nine cells) with a 3 × 3 array over an area of 8 × 8 cm^2^ to address issues in the measurement of tangential force, normal force, and temperature for e-skin applications (Figure 7a) [67]. The researchers proposed a practical and economic method that does not utilize vacuum deposition systems or metal photolithography for sensor fabrication and patterning, and instead uses a printing fabrication technique. Optimal structures that imitate fingerprints enable the simultaneous measurement of normal forces, shear forces, and temperature, as shown in Figure 7b. The results indicate that the developed fully printed flexible three-axial force sensor with temperature sensing components can be used in e-skin applications for the detection of multiple stimuli, despite having a lower spatial resolution and smaller size of the array compared to human skin.

The notable advantages of three-axial force sensors include capabilities in texture sensing in addition to capabilities in direction discrimination. Yeo et al. described the feasibility of wearable mechanotransduced tactile sensors that use highly conductive metallic alloy liquids such as eutectic gallium indium (eGaIn) in a three-dimensional architecture (dome protrusions or bumps) fabricated via soft lithography techniques, extending the range of applications of texture sensing (Figure 7c) [72]. In principle, total electrical resistance is altered by the soft motion of the microfluidic elastomer, in terms of various strains. Figure 7d shows surface profile sensing capabilities on rounded edges, softer materials, and an 80-μm wafer. The researchers explained that electrical resistance fluctuations, sharp peaks, and the smoothness of electrical output profiles in the measurement data are useful for the differentiation of smooth/rough objects and soft/hard objects. However, further studies are required due to the difficulty in comprehensively reading information of surface profiles and converting the information into human perception. Such studies include the analysis of temporal patterns, power spectrum density, and central frequency peaks.

**Figure 8:**
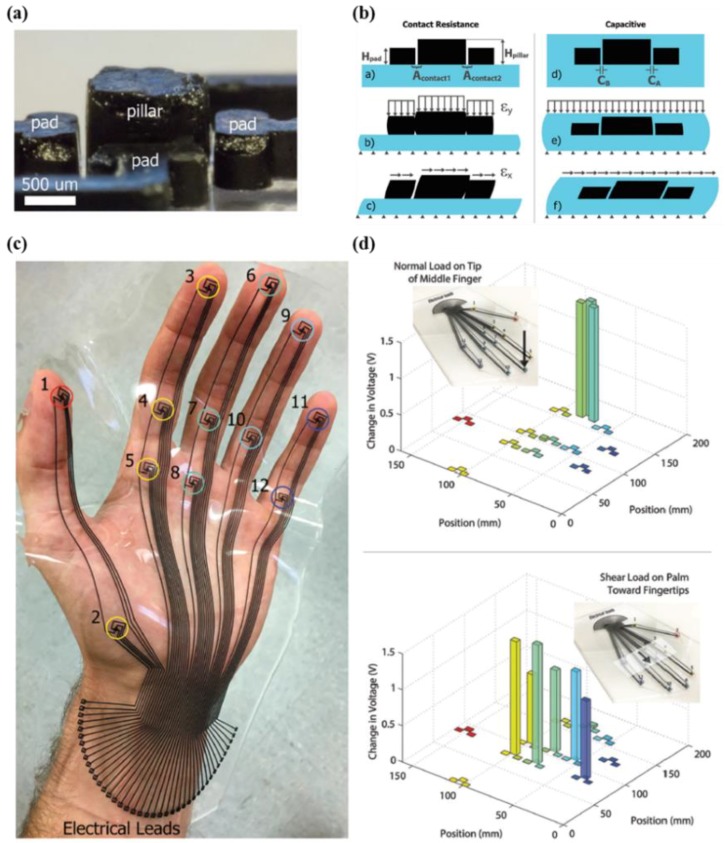
(**a**) Magnified image showing the structure of a unit cell; (**b**) schematic illustration showing the working mechanism; (**c**) photograph of e-skin that fully covers the area of a human hand; (**d**) changes in voltage under normal and shear loads (reprinted with permission for Figure 8a–d from Ref. [71] Copyright 2016 John Wiley and Sons).

Improvements in tactile-sensing capability could lead to a major breakthrough in robotics by enabling dexterous manipulation. In this regard, Charalambides et al. reported the development of three-axial tactile sensors based on all-elastomer materials with mixtures of carbon nanotubes and PDMS (polydimethylsiloxane) for the development of robot skin (Figure 8a) [71]. The researchers employed two transduction methods, which is the contact resistance scheme (normal force range ~8 N, resolution 1 N/shear force range ~400 mN, resolution 100 mN) and capacitive measurement scheme (normal force range ~10 N, resolution 100 mN/shear force range ~1500 mN, resolution 50 mN) for the simple circuit configuration and high dynamic range. The sensor, which was composed of a conductive pillar and pad as shown in Figure 8b, is operated by compression and tension through the Poisson effect. In addition, the researchers obtained experimental data using multidimensional arrays with 12 taxels and 41 electrodes attached to a human hand over a large area, as well as a single cell of a three-axial force sensor (Figure 8c). Figure 8d shows that the developed tactile sensor is capable of mapping normal and shear forces stably with small noise. Using this approach with soft silicone elastomer materials is expected to enable mechanically flexible e-skin with high performance in tactile sensing abilities.

## 4. Novel Materials

### 4.1. Single Crystalline Silicon

The initial step of the dry transfer method (i.e., releasing process for the removal of sacrificial oxide layers using hydrogen fluoride solution) of single crystalline silicon that was improved by John Rogers et al. was developed as a new solution to maintain the superior electrical properties of bulk silicon. The method has contributed to the development of various applications in high-performance electronics [74,75,76], stretchable displays [45], tactile sensors [11,12,13,51,77,78], and biomedical devices [79,80]. Various methods have been investigated for the development of ultrathin single crystalline silicon-based electronics, such as the selective removal of buried oxide using HF [78,81,82,83,84,85,86,87,88,89,90,91,92,93,94,95,96], the bottom etching of silicon substrates using aqueous alkaline solutions of potassium hydroxide (KOH) [97] or tetramethylammonium hydroxide (TMAH), and removal processes using defined ribbon patterns with bridges using reactive ion etching (RIE) [45,98,99]. Conventional electronic devices fabricated using the CMOS (complementary metal–oxide semiconductor) microfabrication processes have drawbacks (e.g., not flexible, fragile in mechanical shock) originating from mechanical properties of bulk silicon, whereas the devices based on ultrathin single crystalline silicon could be mechanically flexible and robust while benefiting from the excellent electrical properties of bulk silicon. A widely used application is in tactile sensors, including silicon strain gauges, which has several advantages in terms of sensitivity (high gauge factor), low hysteresis, easy integration into readout circuitry, good linearity, long-term stability, high reliability, good uniformity, and consistent repeatability [100].

Several studies have strived to develop large-scale integrated networks of sensors by exploiting metal foil strain gauges as well as amorphous and polycrystalline silicon [101,102,103,104,105]. However, such materials face considerable issues due to low sensitivity and limited scalability. In order to overcome these limitations, John Rogers et al. first introduced a piezoresistive strain sensor based on single crystalline silicon nanomembranes on a polyimide substrate [12]. Wheatstone bridge configurations were employed and linked to multiplexing diodes for the calibration of confounding factors affected by temperature change. The gauge factors of the developed heterogeneous system were calculated as 43 for applied strain (0.1%), and 97 for true strain (0.045%) in silicon from finite element modeling (FEM) analysis. Ying et al. also demonstrated finger motion sensors consisting of 4 × 1 arrays of bar-shaped Si-NM strain gauges, serpentine electrodes, and capacitance sensors on the finger tube platform [13]. It is noteworthy that the true strain of single crystalline silicon from FEM analysis was ten times lower than the measured strain, due to the serpentine interconnection lines between neighboring cells.

**Figure 9:**
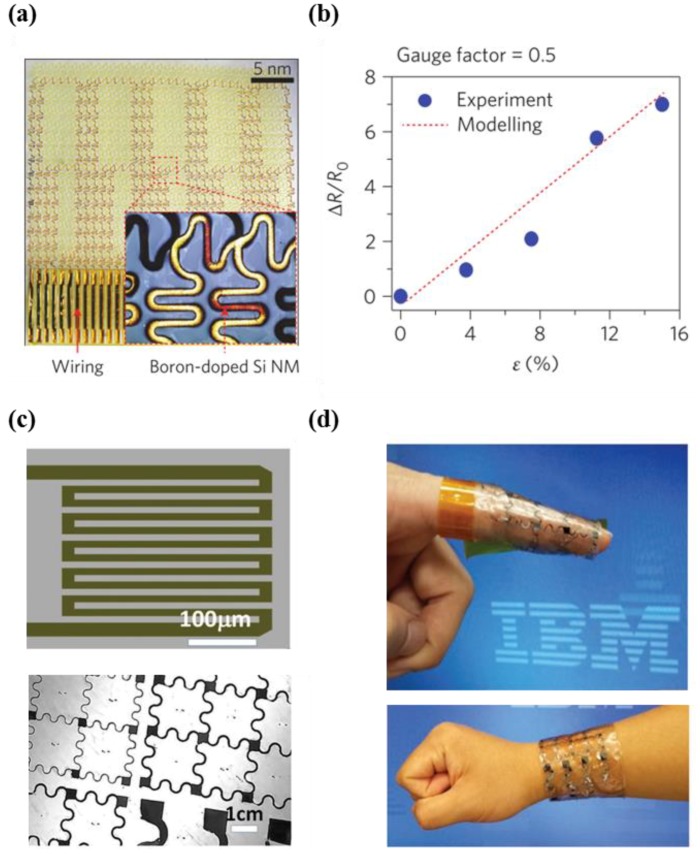
(**a**) Photographic image of silicon strain sensor and location of boron-doped silicon nanomembrane; (**b**) the relative change in resistance versus strain (%) with modeling data (reprinted with permission for Figure 9a,b from Ref. [11] Copyright 2014 Springer Nature); (**c**) image of strain gauge with the serpentine shape pattern and picture of device on tape without stress layer; (**d**) photographic image showing e-skin applications on the finger and wrist (reprinted with permission for Figure 9c,d from Ref. [77] Copyright 2017 John Wiley and Sons).

Son et al. also described a flexible prosthesis that uses single crystalline silicon and gold electrodes with multi-functional sensing capabilities. The developed prosthesis contains strain, pressure, humidity, and temperature sensors, as well as a heater [11]. Figure 9a shows p-doped Si-NMs fabricated via an etching process, which are used to form bar-type and serpentine-type strain gauges. The bar-shape gauge configuration had good output response, despite greater mechanical weakness to strain compared with serpentine-type gauges (Figure 9b). The researchers suggested a cavity structure for high sensitivity, which is commonly utilized in practical silicon diaphragm sensors. However, appropriate solutions should be taken to trade off between durability and sensor performance, as excessive strain (overload conditions) results in cracks or damage in diaphragm pressure sensors.

In recent years, unique and universal layer-release processes (3D spalling) have garnered significant attention as next-generation processes for the realization of high-performance, flexible electronics, due to the limitations associated with the high costs and limited fabrication of releasing processes [77]. Li et al. fabricated a flexible tactile sensor with a serpentine piezoresistor and spring-shaped electrodes on tape via an innovative method using the 3D shape of the final released substrate layer, which is adjusted by the mechanical properties (thickness, stress, Young’s modulus) of a nickel stress layer (Figure 9c). As shown in Figure 9d, the developed sensor possessed good sensitivity with various bending radii, and had a measured gauge factor of 60. The researchers explained that the lower sensitivity of the developed sensor compared with bulk silicon strain gauges (~120) is attributed to the difference in the Young’s modulus between the silicon piezoresistor and the tape.

### 4.2. Graphene

In recent years, significant attention has been placed on graphene materials due to the exceptional optical transmittance (enabling applications in transparent devices) of such materials, as well as the noteworthy mechanical characteristics of graphene materials such as a fracture strain of 25% (enabling highly stretchable electronics) [106,107,108]. This unique class of carbon-based material is expected to open up diverse applications such as transparent electrodes [109,110,111], strain gauge sensors [14,17,112,113,114], high carrier mobility transistors, logic circuits [115,116], touch panels [42,117,118], as well as electrochemical and biomedical devices [119]. This chapter highlights studies of strain gauge sensors that utilize various concepts and growth methods.

**Figure 10:**
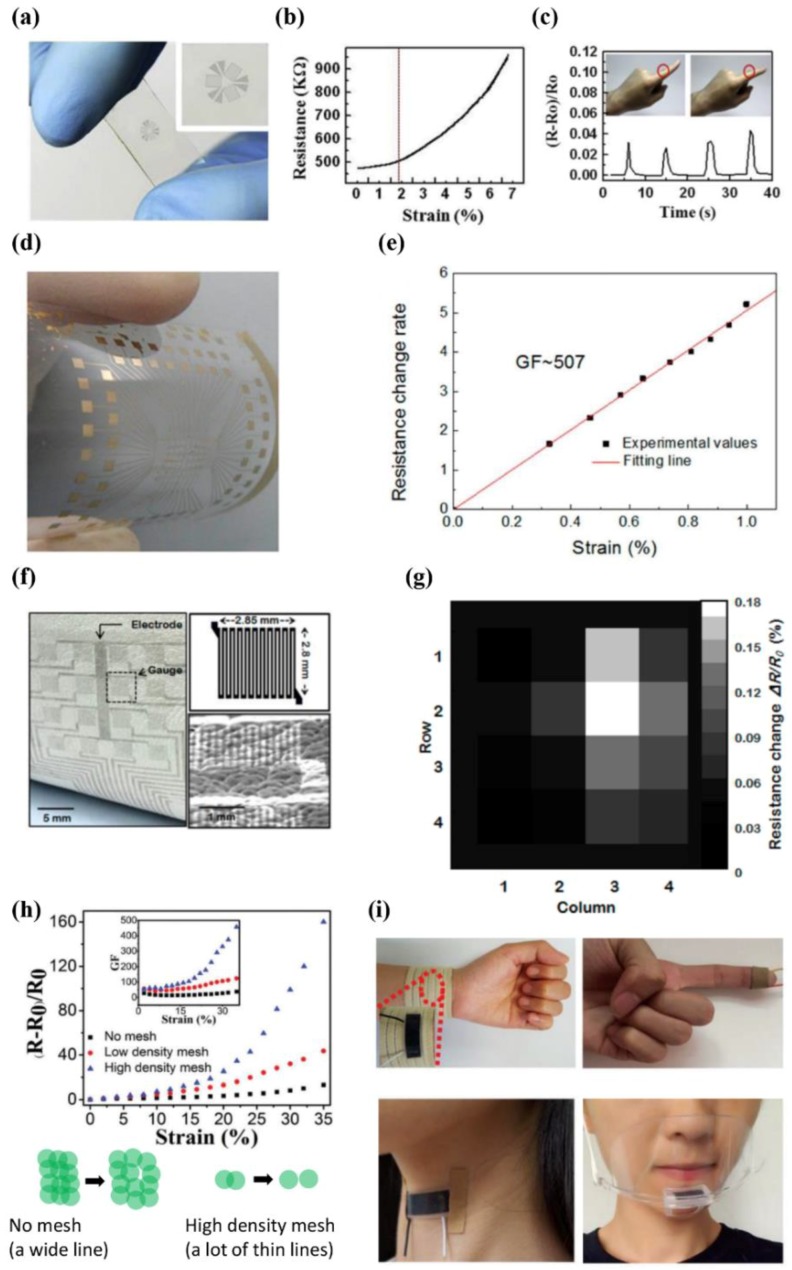
(**a**) Picture showing the transparent graphene strain gauge sensor; (**b**) change in resistance versus stretched strain; (**c**) the relative change in resistance during a stretching experiment of the device on the finger (reprinted with permission for Figure 10a–c from Ref. [14] Copyright 2012 Elsevier); (**d**) photograph of the strain sensor array based on the nanographene film; (**e**) the change in resistance rate with respect to applied strain (reprinted with permission for Figure 10d,e from Ref. [16] Copyright 2015 American Chemical Society); (**f**) photographic and SEM image of graphene strain gauge sensor array on animal leather, the structural information of the unit cell; (**g**) pressure distribution under gentle touch of 9 kPa (reprinted with permission for Figure 10f,g from Ref. [17] Copyright 2014 American Chemical Society); (**h**) output curve variation and the gauge factor values, according to the strain applied to the sensor (top: no mesh, a low-density mesh, and a high-density mesh). The illustration of the working principle (bottom); (**i**) the laser patterned graphene sensor attached onto the wristband, finger, face mask, and throat (reprinted with permission for Figure 10h,i from Ref. [15] Copyright 2017 Royal Society of Chemistry).

Bae et al. demonstrated a graphene-based rosette-type strain sensor with excellent stretchability and good optical transparency (Figure 10a) [14]. The developed graphene strain gauge sensor exhibited two different output characteristics depending on strain value. Below 1.8% strain, the gauge factor was measured as 2.4, which is similar to conventional metallic materials. At strains above 1.8%, the gauge factor was estimated to range from 4 to 14 (Figure 10b). The study explains that this unique performance is caused by variations in effective mass, doping concentration, temperature, crystal imperfections, etc. Figure 10c shows the sensor being applied to detect the bending and straightening movements of a human finger. The results indicate that the developed graphene-based strain sensor can be utilized for future applications in human-interface devices.

Nanographene (NG) that is grown in a remote plasma-enhanced chemical vapor deposition (RPECVD) systems is a promising type of graphene, as CVD-grown graphene has intrinsically low gauge factors due to zero band gap [16]. In particular, high gauge factors of 546 were obtained from two-terminal devices of quasi-continuous NG films developed by Zhao et al. (Figure 10d,e). The researchers of this group explained that the tunneling of charged carriers between adjacent nanographene films contributes to the high piezoresistive effect. The varying nucleation in the NG film affects the tunneling distance, which results in varying sensitivity, with gauge factors ranging from 10 to 10^3^ at similar resistance levels. However, further studies are required to develop larger arrays and smaller sensors, due to the relatively high sheet resistance (10–10^2^ kΩ per square), which renders e-skin applications difficult.

Conformal devices have the benefit of reflecting the movement of skin without mechanical distortion, and are applicable in sophisticated healthcare monitoring [120,121,122], human–machine interfaces [123] and highly sensitive pressure sensors [17,18]. Theoretically, van der Waals forces provide good conformability and enable devices to directly attach to human skin, indicating good conformal coverage. Based on these features, Park et al. developed graphene-based conformal devices such as e-skin and stretchable thin-film transistors [17]. The outstanding performance of conformal ultrathin graphene field effect transistors (UT-GFETs) with a total thickness of 70 nm (atomic-scale graphene, nanometer-thick polymer dielectric films, and a supporting layer of SU-8) on an uneven surface was confirmed. In addition, a piezoresistive graphene strain gauge sensor with a 4 × 4 array was fabricated on animal hide (Figure 10f). The results in Figure 10g indicate that the developed graphene-based conformal tactile sensor is capable of accurately measuring pressure distribution when pressure is applied on the tactile cells.

Graphene strain sensors that are used to measure subtle and large body motions were first fabricated by Tao et al. using the direct laser patterning method [15]. The graphene strain sensor for monitoring subtle motions showed an improved gauge factor of 457 and a strain measuring range of up to 35%, which is optimized to detect small body movements. Especially, the gauge factor of a strain sensor is increased as the mesh density gets higher, since the open state can be formed more easily at a smaller mesh size (Figure 10h). For measurements of large body motions, the strain sensor was tailored to exhibit a slightly lower gauge factor of 268, but an increased detection range of strain up to 100%, which is suitable for obtaining data from large body motions. The researchers described real and commercial applications such as the sophisticated analysis of regular pulse shapes of humans, the monitoring of respiratory systems, and discerning various pronunciations from throat motions (Figure 10i).

### 4.3. MoS_2_

**Figure 11:**
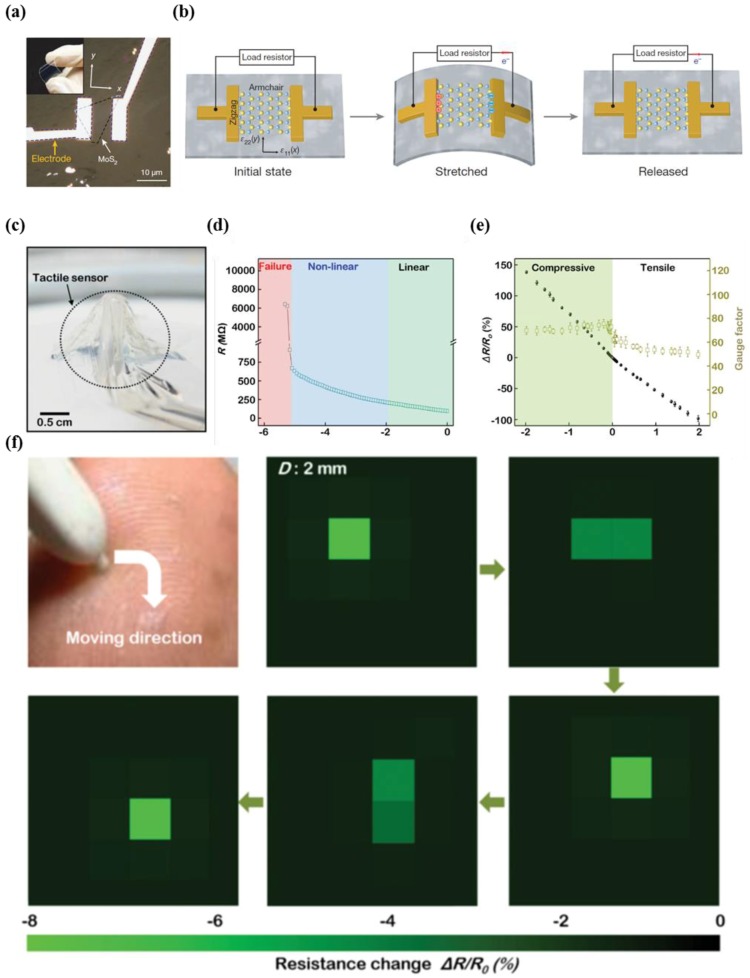
(**a**) Picture and the optical microscopic image of flexible device consisting of single-layer MoS_2_ flake; (**b**) schematic illustration of the operating mechanism of single-layer MoS_2_ piezoelectric device under strain (reprinted with permission for Figure 11a,b from Ref. [137] Copyright 2014 Springer Nature); (**c**) image of conformal tactile sensor based on MoS_2_ floating on the surface of water; (**d**) the output resistance change versus compressive strain; (**e**) fractional change in resistance of the tactile sensor as function of the applied tensile and compressive strain; (**f**) monitoring of pressure distribution (reprinted with permission for Figure 11c–f from Ref. [18] Copyright 2016 John Wiley and Sons).

Transition metal dichalcogenides (TMDs) such as MoS_2_ are attracting interest, as such materials possess comparable or superior characteristics to graphene, including good optical transparency, outstanding mechanical flexibility, and good electrical properties [124,125,126,127,128,129,130,131,132,133,134,135,136]. In particular, ultrathin MoS_2_ semiconductors with high sensitivity and a tunable band gap are strong candidate materials that can be used to produce advanced tactile sensors with good conformability [18]. Wu et al. conducted the first experimental study on the piezoelectric characteristics of two-dimensional MoS_2_, which was grown through seed-free chemical vapor deposition (CVD) (Figure 11a) [137]. After transferring single-domain triangles to flexible PET, the researchers confirmed the piezoelectric current and voltage responses through cyclic stretching and releasing tests, the dependence of charge polarization on the direction of principal strain in two-dimensional (2D) materials, and the evolution of the piezoelectric signal in terms of number of atomic layers in MoS_2_ (Figure 11b). It is noteworthy that MoS_2_ with an odd number of layers exhibited intrinsic piezoelectric properties, which is due to the lack of centrosymmetry in the crystal structure. The estimated gauge factors obtained using the fractional change in current with increasing or decreasing strain was measured as 230 in bilayer MoS_2_, which is considered to exhibit purely piezoresistive properties. The estimated sensitivity is comparable to bulk MoS_2_ devices (~200).

Basing on the exceptional characteristics of MoS_2_, Park et al. first demonstrated an ultrathin conformal, MoS_2_-based tactile sensor array with an area of 2.2 × 2.2 cm^2^ (Figure 11c) [18]. The tactile sensor was fabricated using a photolithographic patterning process, and the devices had a total thickness of less than 75 nm (1.4 nm of MoS_2_, 0.9 nm of graphene, 35 nm of SU-8 passivation layers, and 35 nm of SU-8 substrate). The unit cell of MoS_2_, which had an interdigitated structure (channel width of 37.9 mm and length of 0.05 mm), exhibited three regions: linear (below −1.98% strain), non-linear (from −5.23% strain to −1.98% strain), and failure modes (above −5.23% strain) (Figure 11d). As shown in Figure 11e, the gauge factors were measured as −72.5 ± 1.9 and −56.5 ± 4.8 for compressive strain and tensile strain, respectively. The pressure distributions were recorded in real time when pressure was applied using a stylus pen on the tactile sensor array that was attached to a fingertip. The results in Figure 11f showed that measurements could be effectively conducted during the full e-skin demonstration under pressure, which guarantees the stable operation of e-skin under continuous stimuli of pressure without degradation of sensor performance. The beneficial features could lead to the development of tactile sensors that are capable of discerning multi-directional forces such as normal and shear forces.

## 5. Applications of Tactile Sensing

### 5.1. Multi-Functional E-Skin

Several studies are focused on multi-functional sensors that replicate the tactile sensing capabilities of various human receptors (mechanoreceptors, thermoreceptors, nociceptors) due to the various innovative applications of sensors in humanoid robotics, prosthetic hands/limbs, and haptics for tactile feedback and recording [138,139,140,141,142,143,144,145,146]. Sensors that are capable of measuring various stimuli from the surrounding environment would become a new paradigm in the development of highly intelligent tactile sensing devices.

**Figure 12:**
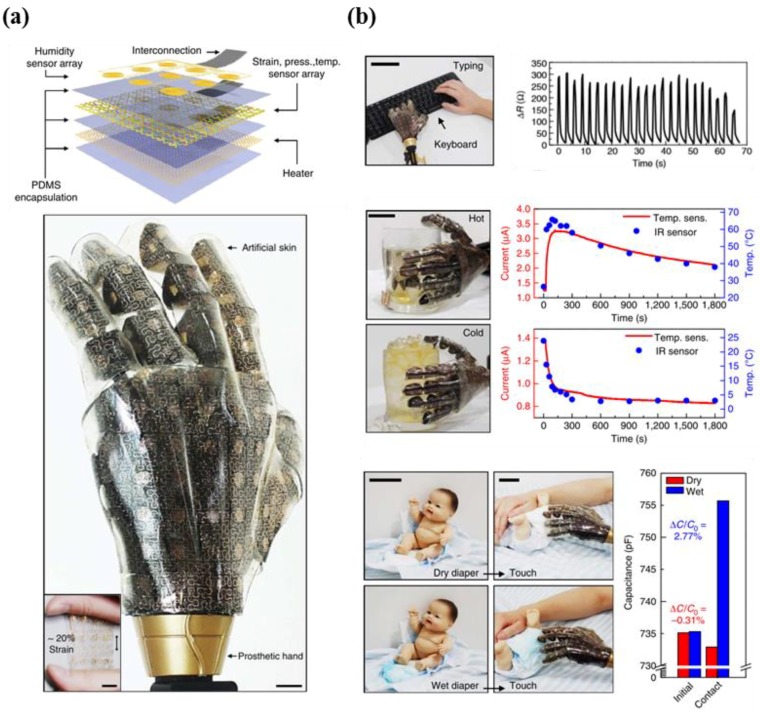
(**a**) Description of the sensor structure (top), image showing the prosthetic hand that is covered with artificial skin. The inset indicates the stretchability of device; (**b**) photograph of tapping keyboard by using prosthetic hand (top left). The changes in resistance of the silicon nanoribbon pressure sensor as a function of time (top right). Photograph of experiment using hot and cold glass (middle left). Real-time monitoring of current change (temperature and IR sensor) (middle right). Image of experimental setup for measuring the humidity sensing capabilities of sensor (bottom left). A bar plot indicating the capacitance change in case of dry and wet states (reprinted with permission for Figure 12a,b from Ref. [139] Copyright 2014 Springer Nature).

Kim et al. introduced a type of stretchable prosthetic skin based on ultrathin single crystalline silicon nanoribbons (SiNR) with strain, pressure, temperature, and humidity sensing capabilities to conduct measurements of external environments, in addition to heating capabilities (Figure 12a) [139]. The researchers adopted strategies for stretchability by using serpentine metal lines and ultrathin layouts for neutral mechanical planes in the SiNR sensor array. This provides good spatio-temporal sensitivity and reliability, as well as wide sensing ranges, which are key in the development of e-skin that mimics human skin. The developed e-skin exhibited outstanding performance in response to various daily life situations such as hand shaking, keyboard tapping, ball grasping, holding a cup of hot/cold liquid, detection of dampness resulting from fluid contact, and contact with humans (Figure 12b).

**Figure 13:**
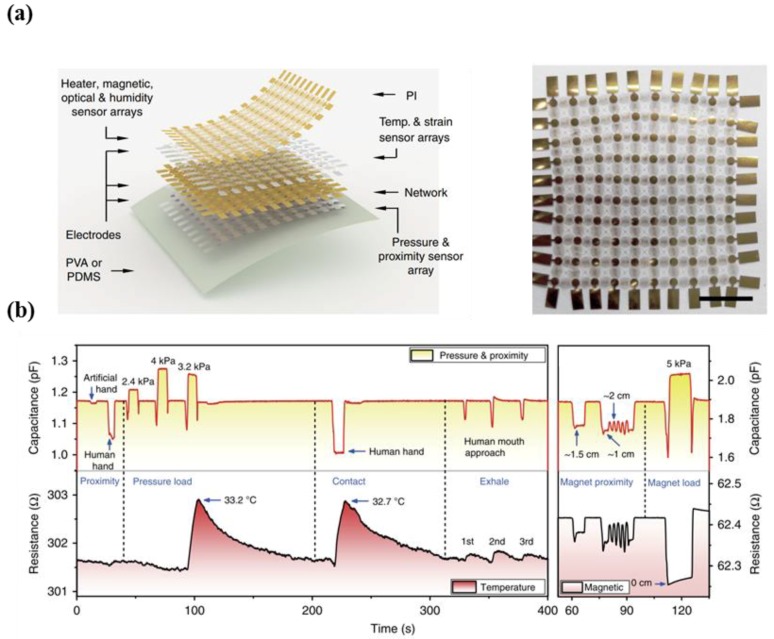
(**a**) Schematic illustration showing the stretchable and conformable matrix networks (left). Photographic image of the device (right); (**b**) sensing capabilities under various multiple stimuli (pressure, temperature, magnetic, and proximity) (reprinted with permission for Figure 13a,b from Ref. [140] under CC-BY 4.0 license).

However, Hua et al. pointed out the limitations of the aforementioned literature [139], as evidence regarding simultaneous measurements of multimodalities was not provided. From this perspective, the research team pioneered the development of a multi-functional e-skin inspired by highly stretchable and conformable matrix networks (SCMN) with various sensing capabilities such as temperature, in-plane strain, relative humidity, UV light, magnetic field, pressure, and proximity (Figure 13a) [140]. In particular, Figure 13b describes the significant features of simultaneous multiple-stimuli sensing capability that differentiates three or more stimuli. Moreover, the researchers stated that the multi-functional e-skin was unique, as it possessed adjustability of sensing range, expandability for large areas, and applicability for high-density three-dimensional integration schemes [140].

**Figure 14:**
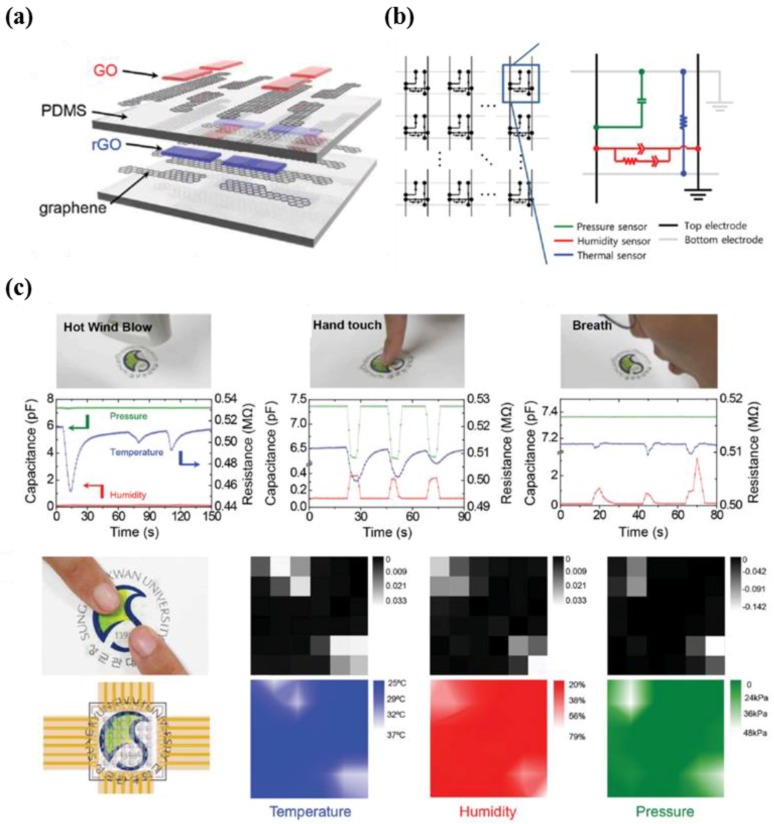
(**a**) Schematic illustration of multimodal E-skin sensor based on graphene material; (**b**) circuit diagram of the device; (**c**) sensing capabilities of the multimodal e-skin sensor corresponding to various stimuli (hot wind blow, hand touch, and breath) (reprinted with permission for Figure 14a–c from Ref. [138] Copyright 2016 John Wiley and Sons).

Ho et al. introduced an all-graphene multi-functional e-skin sensor matrix that was fabricated via a simple lamination process, comprising of graphene oxide (GO)-based impedance humidity sensors, reduced graphene oxide (rGO)-based resistive thermal sensors, and PDMS-based capacitive pressure sensors (Figure 14a,b) [138]. The developed all-graphene e-skin possessed various features such as high optical transparency, good sensitivity, and multimodality to simultaneously differentiate target signals from each sensor. The outputs of the three different types of stimuli, which were hot wind blowing, hand touching, and breathing, show that the responses of the multi-functional e-skin developed in this research was successfully collected (Figure 14c).

**Figure 15:**
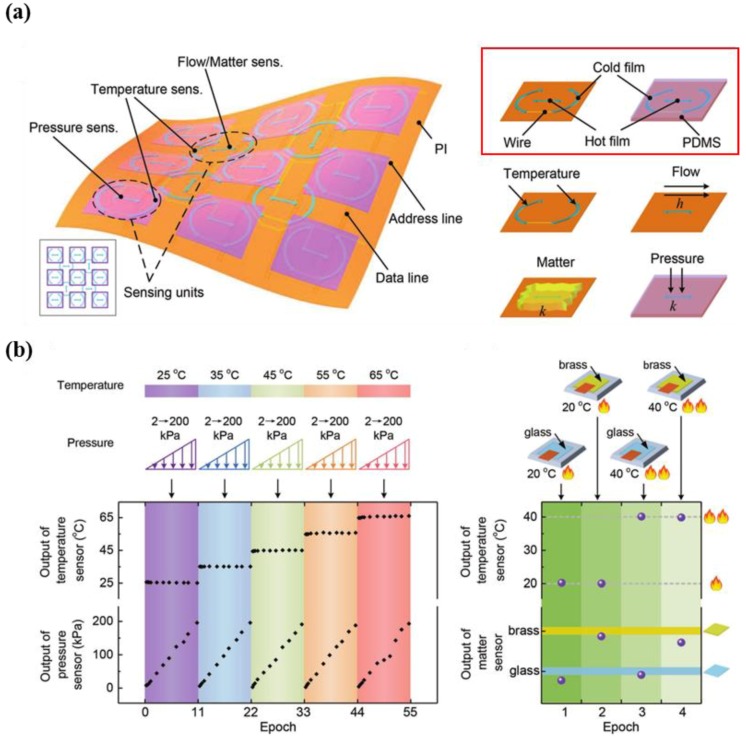
(**a**) Schematic illustration showing the structure of multi-functional e-skin (left) and units (right) including flow, matter, temperature, and pressure sensor; (**b**) multiple performance of the fabricated e-skin (reprinted with permission for Figure 15a,b from Ref. [141] Copyright 2017 John Wiley and Sons).

Zhao et al. developed thermosensation-based artificial skin with multiple sensing capabilities, including the detection of pressure, air flow, matter, and temperature using platinum (Pt) as a sensing material (Figure 15a) [141]. An advantage of this multi-functional device is the simple structure obtained via a patterning process of Pt elements, which enables large-scale and low-cost fabrication. Other advantages include good stability over 7 h and high repeatability during a 2000 cyclic test. The device was subjected to pressures ranging from 2 kPa to 200 kPa under various temperatures ranging from 25 °C to 65 °C. Pressure was applied onto the device under two different temperature values (20 °C and 40 °C) using two different materials (glass and brass), as shown in Figure 15b. Consequently, the results of the multi-parametric e-skin showed simultaneous and selective sensing capabilities in the hybrid situations.

**Figure 16:**
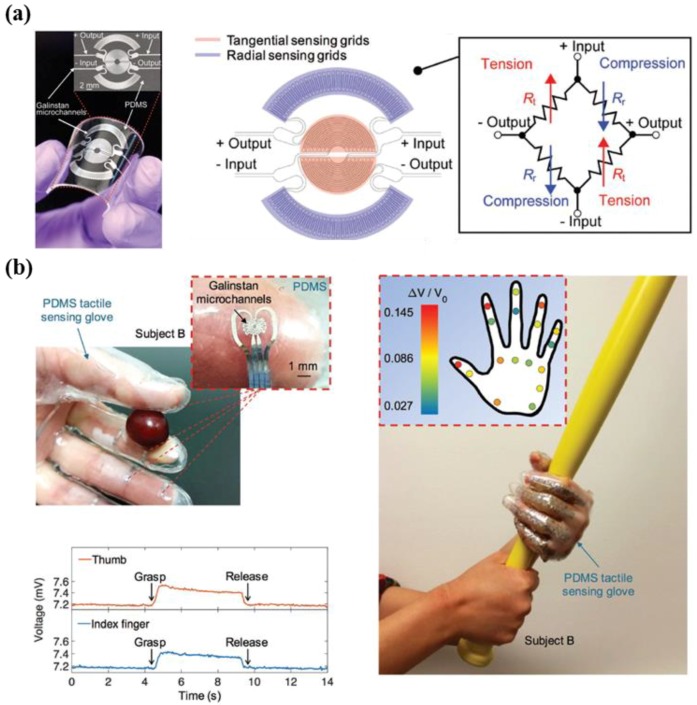
(**a**) Photograph of the microfluidic diaphragm pressure sensor (left). Illustration showing a layout of the sensing area including tangential and radial sensing components (middle). Circuit diagram of the device using a Wheatstone bridge (right); (**b**) Photographic image indicating the tactile sensing glove equipped with the device (top left). Plot showing real-time measurement during the motion of gripping a grape, by using thumb and index finger (bottom left). Photographic image showing the array sensing capabilities, when grasping a bat (right) (reprinted with permission for Figure 16a,b from Ref. [146] Copyright 2017 John Wiley and Sons).

In recent years, many researchers have conducted studies on tactile sensors by using elastic material, such as polydimethylsiloxane (PDMS), due to its benefits, which include high flexibility, biocompatibility, and simple fabrication of micro channels (less than 100 μm), while conventional sensors show drawbacks in inelastic characteristics in active elements [147,148,149]. In addition, liquid state materials are widely utilized, due to their advantages such as good electrical conductivity, non-toxicity, and high deformability [150]. Based on these advantages, Gao et al. explored microfluidic tactile diaphragm pressure sensors that include micro channels of a liquid metal alloy of Galinstan (eutectic alloy of gallium, tin, and indium) with dimensions of 70 μm width × 70 μm height (Figure 16a) [146]. In particular, a pressure sensor with a diaphragm configuration that used a Wheatstone bridge circuit exhibited good performance in terms of linearity, sensitivity (sub-50 Pa, 0.0835 kPa^−1^), resolution (sub-100 Pa), response time (90 ms), and temperature compensation (20–50 °C) (Figure 16b). In addition, the researchers developed a smart glove with multiple pressure sensors using 3D printing molds, as shown in Figure 16c. The results show the various detection capabilities for motions including holding, gripping, grasping, squeezing, lifting, moving, and touch.

**Figure 17:**
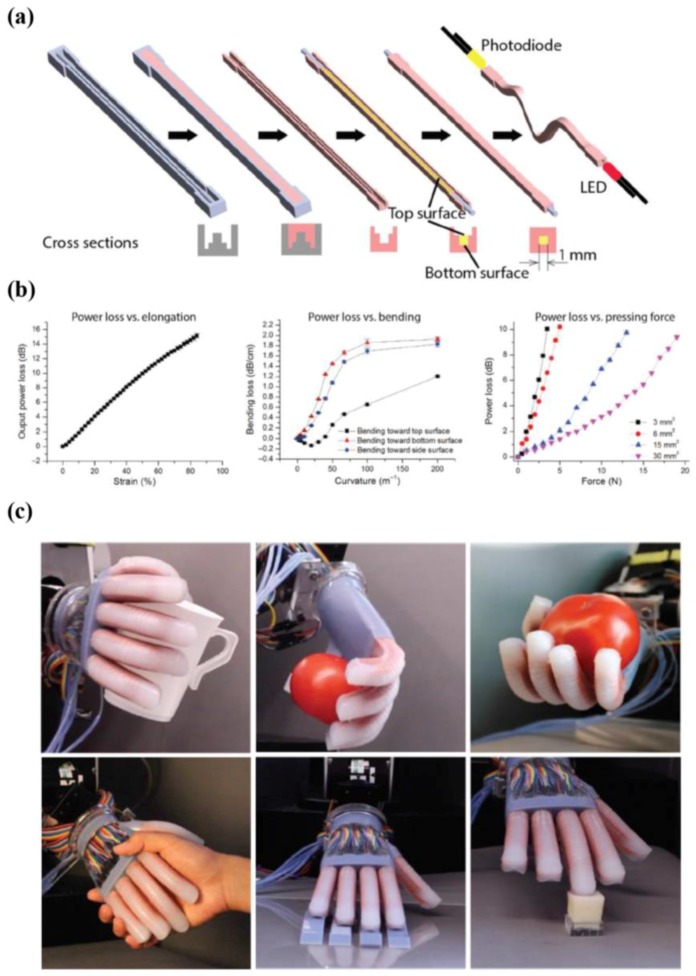
(**a**) Schematic illustration explaining fabrication process of the waveguide; (**b**) output data showing the sensing capabilities of waveguide sensor with respect to different situations including elongation, bending, and pressing force; (**c**) sensing abilities of the device related to the various motions (reprinted with permission for Figure 17a–c from Ref. [142] Copyright 2016 The American Association for the Advancement of Science).

Zhao et al. developed a soft prosthetic hand using stretchable sensors based on elastomeric optical waveguides [142]. The researchers of this group explained that the reported elastomeric waveguides were easily fabricated via soft lithography, in contrast to conventional fabrication processes that require complex and expensive tools (Figure 17a). The core components of the waveguide and cladding are an optically transparent polyurethane elastomer and a highly absorptive silicone composite (ELASTOSIL^™^), respectively. Experimental data regarding the light power loss of the waveguides in the photodiode (power source: a light-emitting diode) was acquired to estimate deformation. The results in Figure 17b show that the output curves of various deformation modes such as elongation, bending, and pressing force have high linearity and repeatability. Lastly, the researchers demonstrated applications for various motions of the prosthetic hand. The soft prosthetic hand showed sophisticated motor skills such as grasping objects, tangential scanning for mapping roughness, and orthogonal control for the detection of softness, as shown in Figure 17c.

### 5.2. Tactile Perception Analysis

Recent achievements associated with the quantification of tactual perception have opened up various potential applications such as highly intelligent robots with a human-like sense of touch, wearable haptic devices that provide real stimuli (virtual reality) [151], and measurement tools for improving tactile aesthetics and experience in the field of cosmetics [152], fabrics [153], and the interior design of automobiles [154,155]. Recent studies have been conducted through two methods: using human fingertips or artificially designed robotic fingertips.

**Figure 18:**
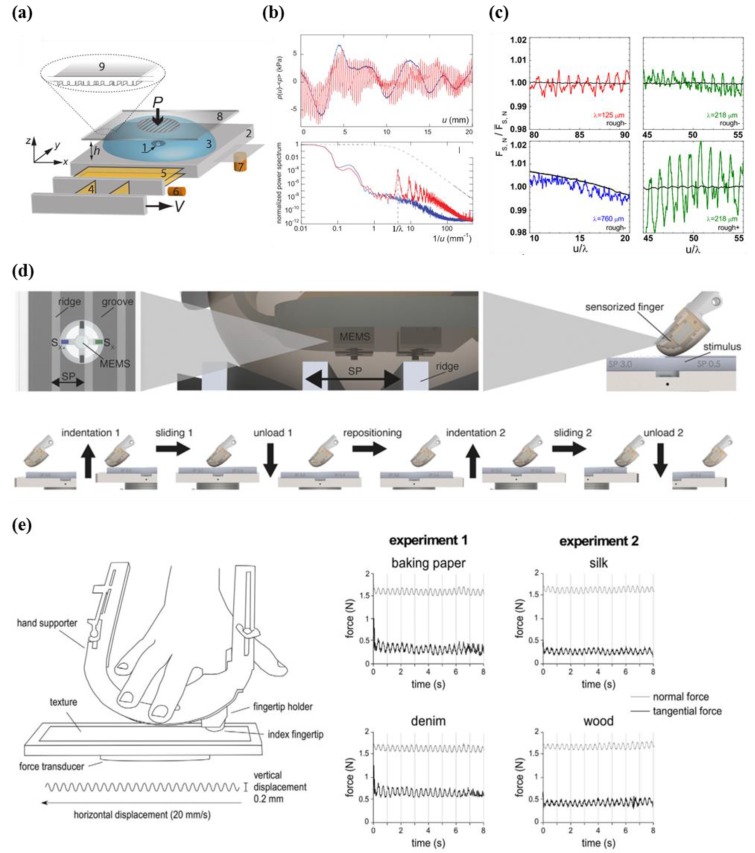
(**a**) Schematic illustration of the measurement setup (left); (**b**) plot of pressure variation according to the displacement (smooth and fingerprinted fingers) (top). Output data of power spectra of signals from Fourier transform (bottom) (reprinted with permission for Figure 18a,b from Ref. [156] Copyright 2009 The American Association for the Advancement of Science); (**c**) plot showing the fluctuations (tangential force) with respect to μ/λ (reprinted with permission for Figure 18c from Ref. [157] Copyright 2011 American Physical Society). (**d**) Schematic illustration showing experimental setup and flow by using sensorized finger (reprinted with permission for Figure 18d from Ref. [9] under CC-BY 4.0 license); (**e**) illustration of experimental setup and tools (left), measured force as a function of time at various texture (baking paper, silk, denim, and wood) (right) (reprinted with permission for Figure 18e from Ref. [160] under CC-BY 4.0 license).

In order to achieve tactual quantification, Scheibert et al. and Wandersman et al. utilized biomimetic MEMS microforce sensors with casting, which mimics a human fingertip (Figure 18a–c) [156,157,158,159]. This approach demonstrated that human tactile perception is closely linked to the frequency spectrum of vibration and a fingerprint-induced modulation of a friction force coefficient that is elicited from the spatial period of fingerprints when textures are rubbed in a horizontal direction (Figure 18d) [9]. However, the incompleteness of skin models results in limitations in comparisons with human skin, in that it is comprised of epidermal ridges, dermis, subcutaneous tissue, nails, fat, and bones. Furthermore, the post-processes correlating to real human perception are highly complex, and researchers cannot analyze mechanical strain in environmental states for the exact evaluation of human tactile perception during exploration without distortion. A direct approach to obtain exploratory movements of a human fingertip involves analyzing the motion of an actual human fingertip (i.e., in situ analysis) [160,161,162,163,164,165,166,167,168,169,170]. Finger-mounted sensors have recently been developed to quantitatively analyze human tactile perception using an acceleration meter (Figure 18e) [160]. However, despite the successful analysis of human tactile perception, accurate quantification is limited by bulky equipment and large amounts of parasitic noise caused by weak adhesions between acceleration sensors and fingertips. For these reasons, researchers are striving to develop a novel tactile sensor for tactile perception evaluation without distortion.

### 5.3. Medical Forcep

Robot-assisted minimally invasive surgery (RMIS) has recently attracted a substantial amount of interest, due to the core idea of using minimally-sized incisions to insert surgical tools into the body of a patient. The technology has several advantages, including reduced intraoperative blood loss, lower risk of postoperative infection, shorter recovery times, and desirable cosmetic effects. However, further studies are required to solve issues such as the lack of haptic feedback, disturbed depth perception, high costs, and poor eye-hand coordination. In particular, haptic feedback is significantly important in preventing tissue damage by excessive force, and offers sophisticated control for the surgeon during the laparoscope operation [171,172,173]. In order to overcome these issues, surgical tools can be equipped with tactile sensors. This chapter introduces recent research related to the integration of tactile sensors in medical tools.

**Figure 19:**
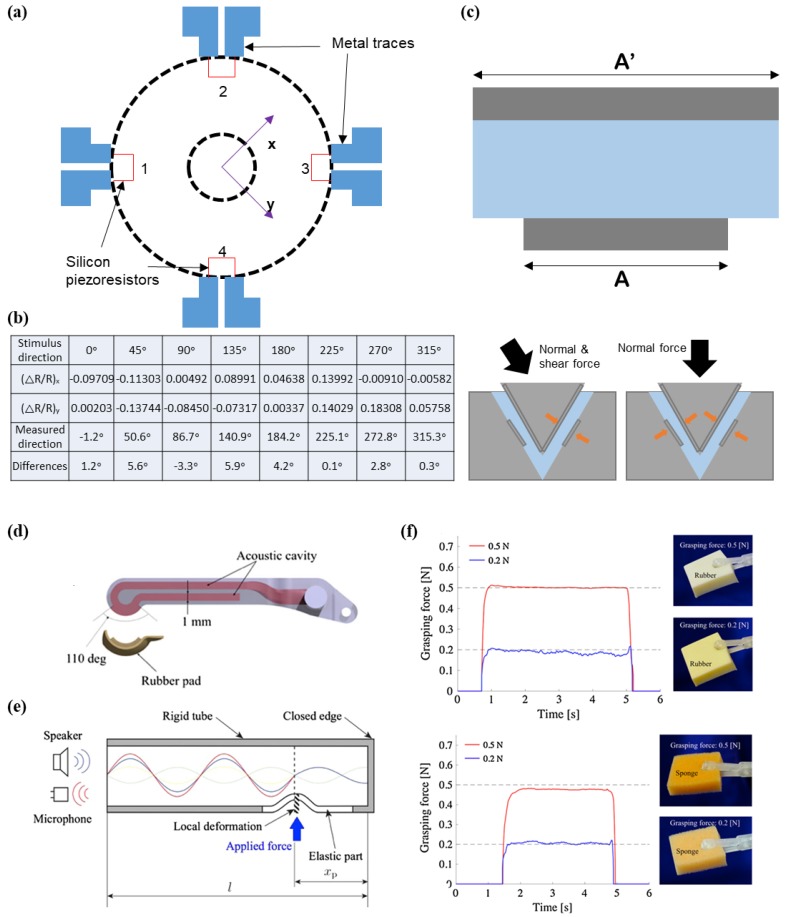
(**a**) Picture of the structure of bio-inspired tactile sensor; (**b**) table indicating data of the scratching test from different force directions measured by the bio-inspired tactile sensor; (**c**) image of the proposed capacitance architecture to measure a three-axis force (top). Illustration demonstrates the sensing mechanism corresponding to normal and shear force (bottom); (**d**) image showing the upper area of forcep with the acoustic cavity; (**e**) diagram indicates the sensing principle of the acoustic tactile sensor; (**f**) experimental data from the acoustic tactile sensor, when grasping the materials, including rubber and sponge (reprinted with permission for Figure 19d–f from Ref. [176] Copyright 2017 Springer Nature).

Hu et al. developed a novel tactile sensor with a center-to-center distance of 1.2 mm between adjacent components, which was inspired by the structure of hair cells. A hair cell is a delicate biological receptor that identifies mechanical stimuli with superb sensitivity. Based on the unique structure of hair cells, the researchers fabricated a tactile sensor comprising of a central silicon column with a height of 470 μm and a thin-film diaphragm with a height of 7 μm from the polyimide substrate at the bottom (Figure 19a) [174]. Four polysilicon piezoresistors are arranged on the surface of the thin-film polyimide as strain gauge sensors. The human hair cell-inspired tactile sensor retains high sensitivity under shear and normal forces, and is thereby able to effectively measure three-dimensional forces. The researchers conducted scratching tests for eight force directions to verify the discrimination performance (Figure 19b). As a result, a small standard deviation of 0.9° was measured during the angle measurement, despite incomplete angle control. In addition to angle measurement, they show continuous output data of normalized resistance changes in the tactile sensor during the scratching tests. The results show that the developed tactile sensor is advantageous in terms of measuring multiple directions. The researchers explained that this sensor is expected to assist surgeons in learning the delicate skills that are associated with haptic feedback in the near future.

Kim et al. introduced an innovative surgical forcep for robotic minimally invasive surgery with haptic feedback [175]. The developed surgical forcep had an integrated four-DOF (degrees of freedom) force sensor, including a three-DOF pulling force and a single-DOF grasping force at the tip of the gripper. In order to sense the four-DOF force, two grasper-integrated capacitive force transducers were embedded in the forceps. The simple design of the sensor was developed with a triangular prism structure, consisting of a capacitance-sensing cell at the tip of the gripper. This capacitive type sensor has a unique design with a different length of the two electrodes, which are in parallel; thereby, the overlapping area (A’ and A) of capacitance is not influenced by horizontal force. Due to this unique design, it can eliminate the effect of horizontal force, and effectively measure both shear and normal force (Figure 19c). The researchers conducted experiments to acquire output data related to pulling and grasping forces in real time. The root mean square (RMS) errors between the sensor and reference sensor, in the x-axis, y-axis, and z-axis directions were 0.08 N, 0.07 N, and 0.11 N, respectively. The results indicate reasonable agreement between the tactile sensor and reference sensor.

Ly et al. developed an innovative grasper with an acoustic cavity to use tactile sensing technology for minimally invasive surgery, by using a sensorized grasper and a single acoustic wave (Figure 19d) [176]. The advantages of this grasper include a suitable area for laparoscopic surgery, simple architecture, simple wiring method, and sterilization performance. The sensor is operated by a difference in the reflection of sinusoidal acoustic wave shapes, in terms of applied force (Figure 19e). Experimental results in Figure 19f show grasping measurement data with various materials such as sponge and rubber, with different grasping forces (0.2 and 0.5 N). The data shows that graspers with tactile sensing capabilities are easily controlled by a user in required surgery environments.

## 6. Conclusions and Perspectives

This paper reviewed the recent advances in tactile sensing technology, focusing on four technical aspects of development: mimicking the tactile sensing mechanisms of humans, the satisfaction of functional requirements of the tactile sensor, the employment of novel materials, and applications in robotic systems and tactile perception analysis. Over the past decade, several research groups have made significant strides in the development of tactile sensors in diverse ways according to the materials used, transducing methods (e.g., analog/digital, piezoresistive, capacitive, or tunneling effect), and special sensing structures (e.g., cracked, dome, or pyramidal-shaped). Such developments provide a variety of viable alternative options for next-generation robotic systems, according to specific applications.

As of this writing, electronic skin that is at the level of human skin has yet to be developed. However, many researchers share the ambition of implementing new tactile sensing technology into robotic prosthetics, medical robots, and humanoid robots, as multimodal sensing is required for accurate interactions with unstructured environments. It seems reasonable to state that most robotic systems will eventually employ tactile sensors along with cameras and microphones for visual and auditory senses, respectively, as the tasks of such robotics systems have changed from repetitive and monotonous work in factories to unplanned and unexpected work in homes (e.g., domestic robots for elderly healthcare services). The main obstacle appears to be technical in nature for the time being. Researchers face major technical challenges that should be addressed in order to meet all of the requirements of the robotics society. For example, a fingertip tactile sensor that is comparable to human skin requires a spatial resolution of 1–2 mm, and should be capable of simultaneously sensing multiple physical quantities with high precision and reliability (e.g., pressure, vibration, temperature, and slip). In addition, the sensors should be wear-proof, chemical-proof, and possess mechanical flexibility and stretchability for conformal attachment onto surfaces with three-dimensional curvatures that are similar to human fingertips. The on-board signal processing units for addressing should be capable of multiplexing, signal conditioning, and analog-to-digital conversion.

Despite such challenges, tactile sensors are expected to continually evolve through incremental improvements to currently available technology, including the various technology described in this review. From a long-term perspective, economics is a factor that is likely to contribute to successful commercialization. Current tactile devices in the market tend to be expensive and highly customized, as the market is not large enough to reap the benefits of high-volume manufacturing. As the market for robots is expected to grow rapidly in size, mass production techniques and innovations in manufacturing techniques should be discovered and developed to render such tactile sensors of research quality practical and convenient for the majority of the robotics developer population. 

## Figures and Tables

**Table 1:** Characteristics of the four main mechanoreceptors in human skin.

	Meissner Corpuscle	Pacinian Corpuscle	Merkel Cell	Ruffini Endings
Classification	RA-I	RA-II	SA-I	SA-II
Adaptation rate	Fast	Fast	Slow	Slow
Location	Shallow	Deep	Shallow	Deep
Stimuli frequency (Hz)	10–200	70–1000	0.4–100	0.4–100
Density (units/cm^2^)	140	20	70	10
Spatial resolution (mm)	3–4	10+	0.5	7+
Functions	Object slip, Light touch, texture	High-frequency Vibrations	Static forces with high resolution	Tension deep in the skin and fascia
Receptive field (RF)	Small and sharp, 3–5 mm	Very large and diffuse, >20 mm	Small and sharp, 2–3 mm	Large and diffuse, 10–15 mm
